# Oncogenic reg IV is a novel prognostic marker for glioma patient survival

**DOI:** 10.1186/1746-1596-7-69

**Published:** 2012-06-19

**Authors:** Qi Wang, Jianping Deng, Jun Yuan, Liang Wang, Zhenwei Zhao, Shiming He, Yongsheng Zhang, Yanyang Tu

**Affiliations:** 1 Department of Experimental Surgery, Tangdu Hospital, Fourth Military Medical University, Xi'an City, 710032, China; 2 Department of Neurosurgery, Tangdu Hospital, Fourth Military Medical University, Xi'an City, 710032, China; 3 Department of Administrative, Tangdu Hospital, Fourth Military Medical University, Xi'an City, 710032, China

**Keywords:** Regenerating islet-derived family member 4, glioma, RT-PCR, Western blot, Immunohistochemistry, Prognosis

## Abstract

**Aim:**

The aberrant expression of regenerating islet-derived family member, 4 (Reg IV) has been found in various human cancers. However, the roles of Reg IV gene and its encoding product in human glioma have not been clearly understood. Therefore, the aim of this study was to investigate the clinicopathological significance of Reg IV expression in glioma.

**Methods:**

Reg IV mRNA and protein expression in human gliomas and non-neoplastic brain tissues were respectively detected by real-time quantitative RT-PCR assay, Western blot, and immunohistochemistry. The association of Reg IV immunostaining with clinicopathological factors and prognosis of glioma patients was also statistically analyzed.

**Results:**

Reg IV mRNA and protein expression levels in glioma tissues were both significantly higher than those in the corresponding non-neoplastic brain tissues (both P < 0.001). Additionally, the increased Reg IV immunostaining in glioma tissues was significantly associated with advanced pathological grade (P = 0.008). Reg IV protein up-regulation was also significantly correlated with low Karnofsky performance score (KPS) (P = 0.02). Moreover, the overall survival of patients with high Reg IV protein expression was dramatically shorter than those with low Reg IV protein expression (P < 0.001). Multivariate Cox regression analysis further confirmed that Reg IV expression was an independent prognostic factor for patients with gliomas (P = 0.008).

**Conclusions:**

These convinced evidences suggest for the first time that Reg IV might accelerate disease progression and act as a candidate prognostic marker for gliomas.

**Virtual slides:**

The virtual slide(s) for this article can be found here:

http://www.diagnosticpathology.diagnomx.eu/vs/2145344361720706

## Introduction

Human glioma represents the most common malignancy in central nervous system for both children and adults. According to the World Health Organization (WHO) classification, gliomas are divided into well-differentiated low grade astrocytomas [WHO grade I ~ II], anaplastic astrocytomas (WHO grade III) and glioblastoma multiforme (GBM, WHO grade IV) [1], which especially represents the most aggressive and the most lethal type of brain tumor. In recent years, there have been great progress occurred in therapeutic technologies, such as surgery, radiotherapy, photodynamic therapy [2,3], and chemotherapy; however, the clinical outcome of glioma patients remains poor, with an average patient life expectancy of only 15 months after diagnosis for GBM patients [4]. The reason for this unfavorable prognosis is that tumor cells in GBM tissues are not only highly proliferative but also readily invade surrounding brain structures, thereby making complete surgical resection practically impossible. Hence, molecular characteristics responsible for the tumor invasion process and the suboptimal response to conventional therapies are potential prognostic and therapeutic markers for human gliomas.

Human regenerating (Reg) genes, initially identified from regenerating rat pancreatic islets, encode five small-secreted proteins (Reg Iα, Reg Iβ, Reg IIIα, Reg IIIβ and Reg IV) with similar structure to the proteins of the calcium-dependent lectin (C-type lectin) superfamily [5]. Reg proteins act as inflammation-modifying acute phase reactants and mucosal repair stimulating factors in the gastrointestinal tract, growth factors and anti-apoptotic survival signal mediators for pancreatic islet cells and neurons [6]. As the most recently discovered member of the human Reg family proteins, Reg IV has been demonstrated to enhance cell proliferation and inhibit apoptosis by inducing phosphorylation of epidermal growth factor receptor (EGFR) (Tyr992, Tyr1068) and Akt (Thr308, Ser473) [7]. In normal human body, the expression of Reg IV mRNA is restricted to gastrointestinal tract, pancreas, prostate, and testes [8]. Normal parietal cells in stomach stain for Reg IV, whereas goblet cells of the ventricular mucosa in areas displaying intestinal metaplasia and in Barrett’s esophagus showed robust accumulation of Reg IV [9]. In normal intestinal mucosa, goblet cells show a weak Reg IV staining, while the mucosa in inflammatory bowel disease is strong Reg IV positive [9]. The aberrant expression of Reg IV has been found in various human cancers, including gastric cancer, colorectal cancer, ductal cancer of the pancreas, gallbladder carcinoma, prostate cancer, and adenoid cystic carcinoma in the salivary gland [10-15]. Recent studies also suggested that Reg IV may play an important role in initiating tumorigenesis, and its detection might be useful in the early diagnosis of tumor formation. However, the role of Reg IV gene and its encoding product in human glioma has not been clearly understood. To address this problem, Reg IV expression at mRNA and protein levels in human glioma and non-neoplastic brain tissues were respectively measured by real-time quantitative RT-PCR assay, Western blot, and immunohistochemistry. The association of Reg IV immunostaining with clinicopathological factors or prognosis of glioma patients was also statistically analyzed.

## Materials and methods

### Patients and tissue samples

This study was approved by the Research Ethics Committee of Tangdu Hospital, Fourth Military Medical University, P. R. China. Written informed consent was obtained from all of the patients. All specimens were handled and made anonymous according to the ethical and legal standards.

A total of 128 formalin-fixed, paraffin-embedded specimens of gliomas resected between 2000 and 2010 were retrieved from the archives of the Pathology Department of Tangdu Hospital, Fourth Military Medical University, P. R. China. All the slides were re-evaluated according to WHO classifications [1] by two pathologists, with differences resolved by careful discussion. A total of 76 males and 52 females (1.46:1) were enrolled in this study, and the median age was 42 years (range, 12–71). Thirty-two of the 128 gliomas were classified as low-grade [18 pilocytic astrocytomas (WHO I) and 14 diffuse astrocytomas (WHO II)], and 96 were classified as high-grade gliomas [38 anaplasia astrocytomas (WHO III), and 58 primary glioblastomas (WHO IV)]. None of the patients had received chemotherapy or radiotherapy prior to surgery. The clinicopathological features and the treatment strategies of all the patients were indicated in Table 1. Paraffin and snap-frozen sections of non-neoplastic brain tissues from 10 patients with intractable epilepsy were also included as controls. Five years follow-up was performed, and all patients had complete follow-up until death. Overall survival time was calculated from the date of the initial surgical operation to death. Patients, who died of diseases not directly related to their gliomas or due to unexpected events, were excluded from this study. In addition, 20 glioma specimens [5 pilocytic astrocytomas (WHO I), 3 diffuse astrocytomas (WHO II), 3 anaplasia astrocytomas (WHO III), and 9 primary glioblastomas (WHO IV)] were snap-frozen in liquid nitrogen and stored at −80°C following surgery for real-time quantitative RT-PCR assay and western blot analysis.

**Table 1:** Clinicopathological features of 128 patients with gliomas

**Features**	**WHO I**	**WHO II**	**WHO III**	**WHO IV**
**Case No.**	18	14	38	58
**Mean age (year)**	38.6	45.9	43.1	44.2
**Gender**				
Male	12	6	25	33
Female	6	8	13	25
**KPS**				
>80	15	11	9	15
<80	3	3	29	43
**Surgery**				
Gross total resection	18	14	28	38
Partial resection	0	0	9	15
Biopsy	0	0	1	5
**Adjuvant treatment**				
Radiotherapy	0	0	30	12
Chemotherapy	0	1	0	6
Radiotherapy and Chemotherapy combination	0	0	5	28

### Real-time quantitative RT-PCR

The mRNA expression of Reg IV in glioma and non-neoplastic brain tissues was detected by real-time quantitative RT-PCR analysis according to the conventional protocols of Tangdu hospital [16]. Especially, the primers were designed as follows: for human Reg IV, forward primer, 5'- TGC ACG ACC CAC AGA AGA G -3', and reverse primer, 5'- GAC TTG CCA GAC CAG GAT CT -3'; for human glyceraldehyde 3-phosphate dehydrogenase (GAPDH), forward primer, 5'- CCC ACT CCT CCA CCT TTG AC-3', and reverse primer, 5'-ATG AGG TCC ACC ACC CTG TT-3'. Each sample was examined in triplicate and the amounts of the PCR products produced were non-neoplasticized to GAPDH which served as internal control.

### Western blot analysis

The protein expression of Reg IV in glioma and non-neoplastic brain tissues was detected by Western blot analysis according to the conventional protocols of Tangdu hospital [16-18]. Briefly, the tissues were harvested by scraping and lysed using modified radioimmunoprecipitation assay buffer (50 mM Tris–HCl, pH 7.4), 1% NP-40, 0.25% sodium deoxycholate, 150 mM NaCl, 1 mM ethylenediaminetetraacetic acid (EDTA), protease inhibitor cocktail complete. The protein content was determined according to Bradford’s method, with bovine serum albumin used as a standard. Equal amounts of protein were separated electrophoretically on 7.5% SDS-polyacrylamide gels and transferred onto polyvinylidene difluoride membranes (Roche; Basel, Switzerland). The membranes were probed with a goat anti-human Reg IV polyclonal antibody (1:25, R&D Systems, Abingdon, UK). The expression level of Reg IV was determined by incubating the membranes with horseradish peroxidase-conjugated anti-goat immunoglobulin G (1:1000 dilution) and enhanced chemiluminescence reagent (Pierce; Minneapolis, MN, USA), according to the manufacturers’ suggested protocols. The membranes were stripped and reprobed with an anti-β-actin mouse monoclonal antibody (1:1000 dilution: Sigma; St Louis, MO, USA) as a loading control. Densitometry was performed using ImageJ software (http://rsb.info.nih.gov/ij/) from National Institutes of Health (NIH; Bethesda, MD, USA). We evaluated the expression of Reg IV as an optical densitometry (OD) ratio that was scored as the densitometry of Reg IV relative to the densitometry of β-actin.

### Immunohistochemistry assay

The immunostaining of Reg IV protein in glioma and non-neoplastic brain tissues was detected by Immunohistochemistry assay according to the conventional protocols of Tangdu hospital [16-18]. Briefly, specimens were blocked with phosphate-buffered saline (PBS) containing 5% normal horse serum (Vector Laboratories Inc., Burlingame, CA, USA) following peroxidase blocking with 0.3% H_2_O_2_/methanol for 30 min. All incubations with goat anti-human Reg IV polyclonal antibody (1:25, R&D Systems, Abingdon, UK) were carried out overnight at 4°C. Then the specimens were briefly washed in PBS and incubated at room temperature with the anti-goat antibody and avidin-biotin peroxidase (Vector Laboratories Inc., Burlingame, CA, USA). The specimens were then washed in PBS and color-developed by diaminobenzidine solution (Dako Corporation, Carpinteria, CA, USA). After washing with water, specimens were counterstained with Meyer’s hematoxylin (Sigma Chemical Co., St Louis, MO, USA). Non-neoplastic brain tissues were used as control tissues and non-immune IgG was also used as negative control antibody for immunohistochemical staining. The immunohistochemical reaction was performed simultaneously for all slides.

Assessment of immunohistochemical staining was evaluated by two independent pathologists. The scores of the two pathologists were compared and any discrepant scores were trained through re-examining the stainings by both pathologists to achieve a consensus score. The number of Reg IV positive-staining cells showing immunoreactivity in ten representative microscopic fields was counted and the percentage of positive cells was calculated. The percentage scoring of immunoreactive tumor cells was as follows: 0 (0%), 1 (1-10%), 2 (11-50%) and 3 (>50%). The staining intensity was visually scored and stratified as follows: 0 (negative), 1 (weak), 2 (moderate) and 3 (strong). A final immunoreactivity scores (IRS) was obtained for each case by multiplying the percentage and the intensity score. The median of IRS for all examined samples was 5.3. Thus, the Reg IV protein expression levels were further analyzed by classifying IRS values as low (based on a IRS value less than 5.3) and as high (based on a IRS value greater than 5.3).

### Statistical analysis

All computations were carried out using the software of SPSS version13.0 for Windows (SPSS Inc, IL, USA). Data were expressed as means ± standard deviation (SD). The analysis of variance (ANOVA) was used to determine the statistical differences among the groups. Spearman rank correlation is used to analyze the correlation between Reg IV mRNA expression and Reg IV protein expression. A life table was calculated according to the Kaplan-Meier method. Hazard ratios for the time-to-event endpoint were estimated using the multivariate Cox regression analysis in a forward stepwise method to evaluate the effect of multiple independent prognostic factors on survival outcome. Differences were considered statistically significant when *p* was less than 0.05.

## Results

### Reg IV mRNA and protein expression in human glioma tissues

**Figure 1:**
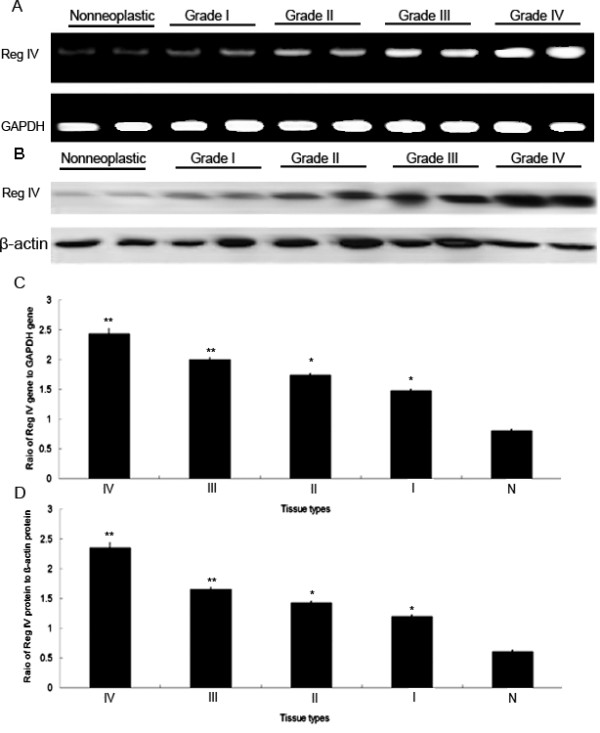
**Reg IV mRNA and protein expression in 20 glioma tissues in different grades and non-neoplastic brain tissues were respectively detected by real-time quantitative RT-PCR assay and Western blot analysis.** (**A**) Expression levels of Reg IV mRNA in glioma tissues with different grades and non-neoplastic brain tissues. (**B**) Expression levels of Reg IV protein in glioma tissues with different grades and non-neoplastic brain tissues. (**C**) A graphical representation of the Reg IV mRNA level expression profiles in (A). (**D**) A graphical representation of the Reg IV protein level expression profiles in (B). 'N' refers to non-neoplastic brain tissues; 'I ~ II' refers to glioma tissues with grade I ~ II; 'III ~ IV' refers to glioma tissues with grade III ~ IV. *P < 0.01, compared with ‘N’; ‘**’P < 0.001, compared with ‘N’.

The expression levels of Reg IV mRNA were detected in 20 glioma and 10 non-neoplastic brain tissues normalized to GAPDH. As shown in Figure 1A and C, the expression levels of Reg IV mRNA were found to be distinctly increased in glioma tissues compared to non-neoplastic brain tissues, corresponding to the glioma WHO grades. The statistic results showed that its expression in high-grade (III: 2.4 ± 0.1; IV: 2.0 ± 0.09) and low-grade (I: 1.5 ± 0.03; II: 1.7 ± 0.07) gliomas were both significantly higher than that in non-neoplastic brains tissues (0.8 ± 0.03; Grade I *vs.* N: P = 0.003; Grade II *vs.* N: P = 0.003; Grade III *vs.* N: P < 0.001; Grade IV *vs.* N: P < 0.001; Figure 1C). Additionally, there was also a significant increasing in mRNA copies of Reg IV corresponding to the glioma WHO grades (P = 0.002). With the agreement of above results, Western blot analysis also found Reg IV protein expression was 2.3 ± 0.1, 1.6 ± 0.08, 1.4 ± 0.06, 1.2 ± 0.03, 0.6 ± 0.01 for glioma grade IV, glioma grade III, glioma grade II, glioma grade I and non-neoplastic brain (N), (Grade I *vs.* N: P = 0.004; Grade II *vs.* N: P = 0.004; Grade III *vs.* N: P < 0.001; Grade IV *vs.* N: P < 0.001; Figure 1B, D). Notablely, the Reg IV mRNA expression was closely correlated with its protein expression (r = 0.7; P = 0.01).

### Reg IV immunostaining and its association with clinicopatholigcal features of gliomas

**Figure 2:**
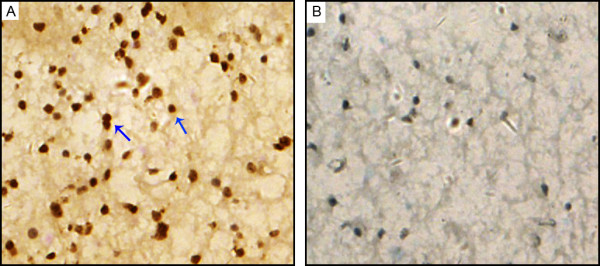
**Representative sections for Reg IV immunoreactivity in glioma tissues (A) and nonneoplastic brain tissues (B) (×400).** Reg IV was mainly expressed in the nuclei with brown yellow.

Reg IV protein expression and cellular localization were also detected by immunohistochemistry assay in 128 glioma specimens and 10 non-neoplastic brain tissues. In all non-neoplastic brain tissues, no Reg IV positive staining was found (Figure 2A), whereas Reg IV positive staining was localized in the nuclei of tumor cells in glioma tissues (Figure 2B). The cellular localization of Reg IV protein found in this study was consistent with the previous studies of Oue *et al.*[12], HAYASHI *et al.*[13] and Tamura et al. [14]. Of 128 glioma specimens, 106 (82.8%) highly expressed Reg IV. Then, we analyzed the associations of Reg IV expression with various clinicopathological parameters of glioma tissues. Data in Table 2 showed that the high level of Reg IV expression was significantly more common in glioma tissues with advanced pathologic grade than those with low pathologic grade (P = 0.008). Especially, the frequencies of Reg IV high expression in glioma tissues with high pathologic grades were more than those with low pathologic grades (Grade III: 84.2% *vs.* 15.8%, P < 0.001; Grade IV: 100.0% *vs.* 0, P < 0.001). In addition, a significant relationship was also found between Reg IV expression and the KPS. Increased expression of Reg IV protein more frequently occurred in tumors with low KPS than those with high KPS (P = 0.02, Table 2). However, there was no significant association between Reg IV expression and other clinicopathological parameters, including gender and age at diagnosis (P > 0.05, Table 2).

**Table 2:** Association of Reg IV expression in human glioma tissues with different clinicopathological features

**Clinicopathological features**	**No. of cases**	**Reg IV expression**		**P**
		**High (n, %)**	**Low (n, %)**	
**WHO grade**				
I	18	8 (44.4)	10 (55.6)	0.008
II	14	8 (57.1)	6 (42.9)	
III	38	32 (84.2)	6 (15.8)	
IV	58	58 (100.0)	0 (0)	
**Age**				
<55	52	42 (80.8)	10 (19.2)	NS
≥55	76	64 (84.2)	12 (15.8)	
**Gender**				
Male	76	64 (84.2)	12 (15.8)	NS
Female	52	42 (80.8)	10 (19.2)	
**KPS**				
<80	78	72 (92.3)	6 (7.7)	0.02
≥80	50	34 (68.0)	16 (32.0)	

### Prognostic value of reg IV expression in overall survival of patients with gliomas

**Figure 3:**
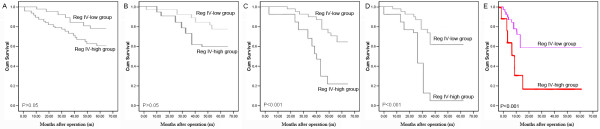
Kaplan-Meier survival curves for glioma patients in WHO grade I (A), grade II (B), grade III (C), grade IV (D), and all patients (E) with high and low expression of Reg IV.

In order to investigate the prognostic value of Reg IV expression in overall survival of patients with gliomas, the detail clinical information of all 128 gliioma patients in Reg IV-high or -low groups was reviewed. During the follow-up period, 100 of 128 glioma patients (78.1%) had died [90 (84.9%) from the Reg IV-high group and 10 (45.5%) from the Reg IV-low group]. We evaluated the prognostic significance of Reg IV protein expression levels in different subgroups of glioma patients stratified according to the WHO grading. Notably, high Reg IV expression also significantly correlated with shorter overall survival time in subgroups of glioma patients with grade III and grade IV (both P ≤ 0.001, Figure 3C and D). However, the overall survival of glioma patients in grades I and II subgroups had no significant differences between high Reg IV expression and low Reg IV expression (P ≥ 0.05; Figure 3A and B). Moreover, as determined by the log-rank test, the survival rate of all glioma patients with high Reg IV expression was significantly lower than those with low Reg IV expression (P ≤ 0.001; Figure 3E).

In multivariate analysis, Cox proportional hazards model involving the expression level of Reg IV protein and various clinical parameters identified the high Reg IV protein expression (P = 0.008) as an independent prognostic factor for glioma patients. Statistical values of the expression of Reg IV and other clinical parameters derived from Cox stepwise proportional hazards model were indicated in Table 3.

**Table 3:** Cox multivariate analysis

**Parameter**	**Risk ratio**	**95% confidence interval**	**P**
**Age**	0.9	0.6-1.7	0.7
**Gender**	1.0	0.7-1.8	0.3
**KPS**	2.0	1.3-3.0	0.1
**Extent of resection**	1.3	0.9-2.1	0.1
**Type of adjuvant treatment**	1.4	1.0-2.2	0.1
**Reg IV expression**	6.9	1.1-18.3	0.008

## Discussion

Understanding of the molecular alterations that occur during tumorigenesis, and identification of novel markers for cancer diagnosis and novel targets for treatment, may be important for the improvements in tumor diagnosis, treatment and prevention. This study was focus on human glioma, which is an aggressive tumor with heterogeneous tumor biology, high invasiveness, rapid tumor cell proliferation and poor prognosis. Several genetic and molecular mechanisms of tumorigenesis in gliomas have been explained; however, factors affecting this tumor progression remain unclear. As results of our analysis, there are four points of findings. Firstly, Reg IV was up-regulated in human glioma tissues compared with non-neoplastic brain tissues at both mRNA and protein levels; Secondly, the increased Reg IV expression in glioma tissues was significantly correlated with advanced tumor progression and aggressive clinicopathological features; Thirdly, the results of Kaplan-Meier analyses shown that glioma tissues with high Reg IV expression tend to have poorer overall survival. Finally, the multivariate analysis clearly demonstrated that the high expression of Reg IV was a statistically significant risk factor affecting overall survival in glioma patients, suggesting that Reg IV expression could be a valuable marker of glioma progression and prognosis. To our knowledge, this is the first study to analyze the expression patterns and clinical significance of Reg IV at transcriptional and translational levels in a large number of glioma patients.

Reg genes encode five multifunctional small-secreted proteins, which can act as acute phase reactants, lectins, or antiapoptotic or growth agents [19]. As a novel member of the Reg family, human Reg IV is mapped to chromosome 1q12-q21, whose cDNA contains an open reading frame of 477 bp encoding a peptide of 158 amino acids with a predicted molecular mass of 18 kD [20]. Reg IV functions as a potent activator of the EGFR/Akt/AP-1 signaling pathway in several types of cancer cells and increases the expression of Bcl-2, Bcl-xl, and surviving proteins, related to the inhibition of apoptosis, suggesting that it may act as a tissue mitogen or play a role in the cell growth [21]. Additionally, Reg IV has been found to be colocalized with Ki-67, indicating that it may be involved in the proliferative process of epithelial cells [22]. Besides this, it also contributes to liver metastasis by inducing the expression of matrix metalloproteinase 7 [23]. In recent year, accumulating studies on Reg IV had reported its presence and overexpression in numerous cancers. The up-regulation of Reg IV in colorectal adenomas was testified by several different research groups [10,24,25]; Zhang *et al.*[24] also indicated that Reg IV might play an important role in initiating colorectal adenoma, and its detection might be useful in the early diagnosis of colorectal adenoma formation. In gastric carcinogenesis, Zheng *et al.*[26] detected the increased expression of Reg IV from gastric intestinal metaplasia to adenoma, but the decreased expression during the malignant transformation of gastric epithelial cells. These findings revealed that Reg IV expression should be considered as a good biomarker for gastric precancerous lesions. In prostate cancer, Hayashi *et al.* found the higher serum Reg IV concentration compared with control individuals, indicating that serum Reg IV represents a novel biomarker for this disease [15]. In line with these previous studies, our data in the present study demonstrated the up-regulation of Reg IV mRNA and protein in human glioma tissues. Interestingly, we also determined that the Reg IV mRNA expression was closely correlated with its protein expression, suggesting that the up-regulation of Reg IV in gliomas may be primarily caused by transcriptional activation. Additionally, its overexpression was associated with advanced pathological grade and low KPS. These results confirmed the contribution of Reg IV expression to the aggressive progression of human gliomas.

Given above intriguing observation correlating Reg IV up-regulation with advanced tumor progression in human gliomas, we were further interested in studying a potential prognostic value of Reg IV for this malignancy. As the results, overall survival after surgical resection among glioma patients in Reg IV-high group was poorer than those in Reg IV-low group. However, there are some different findings on the relationship between Reg IV expression and survival of patients with cancer. For example, Tamura *et al.*[14] revealed that the positive Reg IV expression might be independently associated with a favorable prognosis in patients with advanced gallbladder carcinoma. In gastric cancer, no statistically significant prognostic effect of Reg IV was found [25]. These different reports suggest that the contribution of Reg IV expression to the clinical outcome of patients may be different according to the type of malignancy.

In conclusion, our data offer the convince evidence for the first time that Reg IV might accelerate disease progression and act as a candidate prognostic marker for human gliomas. Further elucidation on the roles of Reg IV as tumor promoter in gliomas is worth to be done.

## Competing interest

The authors declare that they have no competing interest.

## Authors’ contributions

QW dealt with the samples. JD, LW, ZZ and SH provided the samples collected patients information. JY and YZ drafted the manuscript. YT designed the project. All authors read and approved the final manuscript.
